# The Pandemic Experience: A Corpus of Subjective Reports on Life During the First Wave of COVID-19 in the UK, Japan, and Mexico

**DOI:** 10.3389/fpubh.2021.725506

**Published:** 2021-08-20

**Authors:** Tom Froese, Matthew Broome, Havi Carel, Clara Humpston, Alice Malpass, Tomoari Mori, Matthew Ratcliffe, Jamila Rodrigues, Federico Sangati

**Affiliations:** ^1^Embodied Cognitive Science Unit, Okinawa Institute of Science and Technology Graduate University, Okinawa, Japan; ^2^School of Psychology, University of Birmingham, Birmingham, United Kingdom; ^3^Department of Philosophy, University of Bristol, Bristol, United Kingdom; ^4^Bristol Medical School, University of Bristol, Bristol, United Kingdom; ^5^Human Subjects Research Review Committee, Okinawa Institute of Science and Technology Graduate University, Okinawa, Japan; ^6^Department of Philosophy, University of York, York, United Kingdom

**Keywords:** COVID-19, subjective reports of experience, social distancing, corpus, cross-cultural survey

## 1. Background

The first cases of COVID-19 were reported in Wuhan, China, in December 2019, and a month later the Emergency Safety Committee of the International Health Regulations officially declared the situation as a Public Health Emergency of International Importance. The virus quickly spread worldwide and in March 2020 was declared a pandemic. Until now, nearly two-hundred million cases of infection have been confirmed, causing nearly four million deaths. The pandemic and its consequences are having a disastrous impact on societies around the world, and this protracted global crisis is reflected in people's experience (1). Importantly, the mental health of many people across the world has been affected (2). Even in many healthy individuals feelings of fear, uncertainty, distrust, and loneliness are more common, as are raised stress and anxiety levels (3), and this situation may aggravate symptoms for those with pre-existing mental health conditions (4). Many are grieving the loss of their loved ones, and more generally a loss of meaning in life (5).

At the same time, there is also notable heterogeneity in how people have responded to this crisis; many have showed surprising levels of resilience (6), for example by turning to technology-based social interactions to compensate for lockdown restrictions (7). It is therefore crucial to combine such population-level assessments with a more individual-centered approach.

Historical archives show us that past pandemics were relatively poorly documented, but this time around there are widespread efforts to keep a detailed record (8). Our main concern, as a multidisciplinary team spanning psychology, philosophy, psychiatry, medicine, and anthropology, is that this record also includes a detailed account of how people experienced the pandemic from their own first-person perspective. We therefore decided to publicly release a cross-cultural corpus of subjective reports of the first wave of COVID-19.

There is a growing number of cross-cultural investigations of how people were impacted by the pandemic, but they usually focus on specific segments of the population and/or address very specific research questions, and often in terms of quantitative scales rather than in the form of subjective reports [e.g., (9–12)]. The value of this corpus is that it was open to all adults, and that it was intended to capture people's first-person perspective on how they experienced the pandemic impacting on various essential aspects of their everyday life. It achieved this by asking participants to describe their experiences in their own words in response to a series of thematically organized questions that were carefully crafted to facilitate this process of reflection, by drawing on the authors' extensive expertise in relevant areas such as phenomenological philosophy, phenomenological psychopathology, and enactive cognitive science [e.g., (13–16)].

The publicly accessible link to the online survey was distributed via the authors' social networks, news releases by their academic institutions, publications, presentations, as well as via Facebook advertising campaigns that was targeted specifically at residents in UK, Japan, and Mexico. We focused on those three countries in particular in order to capture subjective reports from participants broadly representative of the distinct sociocultural regions of Asia, Europe, and Latin America. Although COVID-19 has caused a global crisis, the way countries, societies, and communities deal with the crisis is also importantly local and context-specific. For example, depending on where you live, a greeting between friends can involve different degrees of bodily contact, e.g., kissing and hugging, a handshake, or bowing, and these practices will be more or less affected by social distancing requirements.

This is not the place to provide a systematic cross-cultural comparison of these regions, so we only offer very general indications to orient the reader. According to Hofstede (17), culture in Mexico, Japan, and the UK varies along several dimensions. For example, interpersonal communication in Mexico and Japan is generally less verbally explicit, and yet decisions and activities often revolve around personal, face-to-face relationships. The UK, on the other hand, is known for the directness of communicative practices. Thus, the corpus enables researchers to investigate what are culture-specific, in contrast to culture-independent, aspects of the pandemic experience, for example by comparing Japanese-language participants, whose socio-cultural practices tends to be regarded as indirect, modest, and collective, with English-language participants, who are known for a more direct, goal-oriented, and individualistic society.

In order to contextualize the responses of the participants, it helps to get a sense for the pandemic situation in the three representative countries during the time of data collection. According to Our World in Data (18), at the start of the survey, on June 5, 2020, the UK, Japan, and Mexico had a cumulative total of 264,150, 16,958, and 110,026 confirmed cases of COVID-19, respectively. And on that day alone the three countries reported 1,255, 41, and 3,628 new cases, as well as 170, 4, and 536 deaths due to COVID-19, respectively. In total, for the 2-month period of June 5–July 31, 2019, the UK, Japan, and Mexico reported 40,650, 19,272, and 314,614 new cases, and 2,769, 92, and 33,518 deaths, respectively.

All three countries had varying degrees of social distancing measures in place for specific regions, so a general picture is difficult to obtain. However, it seems that the severest lockdown measures that had been in operation since March 2020, were lifted around the start of the survey. For instance, in the UK in the first week of June non-essential shops were allowed to open again, albeit with social distancing in place; face masks were made mandatory the following week. In the case of Mexico, Mexico City was taken out of lockdown from mid-June onward. Japan's state of emergency was over by May 25, 2020 for all prefectures and was not reinstated for the duration of the survey. The survey therefore captures the time period when people had recently started coming out of the most severe social distancing measures, and were slowly adapting to the “new normal” of living with the pandemic under moderate restrictions.

## 2. Methods

We ran the survey on the online platform SurveyMonkey from June 5 to July 31 2020. Participant recruitment was primarily from the authors' social media networks and Facebook, which consisted of five Facebook advertising campaigns launched in the UK, Mexico, and Japan during the survey period. The minimum age to participate in the survey was 18.

It received 2,543 responses. Some responses did not meet the criteria for inclusion—59 participants did not grant their consent to participate, 681 participants did not fill out their name and email and hence did not proceed to the rest of the survey, and one participant was underage. Hence, 1,801 participants fulfilled the criteria, and their responses are included in the corpus. Out of the 1,801 participants that were included, 1,694 answered at least one open question. The resulting total word count for the entire corpus is 574,051 words with 2,732,007 characters. The final breakdown of respondents by language was: 1,051 English, 507 Spanish, and 243 Japanese respondents. All responses were translated into English by professional translators, and original language responses are also included in the corpus.

It should be noted that because this was an open survey available on the Internet, overall participants were demographically more diverse, with participants from 55 different nationalities and 51 different countries of residence and with ages that spanned from 18 to 90. However, here we focus our analysis on the specifically targeted countries of UK, Japan, and Mexico. More detailed demographic data corresponding to English, Japanese, and Spanish responses can be found in Table 1.

**Table 1:** Statistics and demographics of the Pandemic Experience corpus.

	**English**	**Japanese**	**Spanish**	**Total**
Total responses	1,376	434	733	2,543
Selected responses	1,051	243	507	1,801
Responded to at least 1 open-ended question	983	243	467	1,693
Word count	409,767	4,830	159,454	574,051
**Gender**				
Male	247	106	185	538
Female	730	125	274	1,129
Other	16	1	10	27
**Age**				
Range	18-87	18-87	18-81	18-87
Median	53	52	40	50
**Country of residence**				
UK	760	0	1	761
Mexico	19	0	424	443
Japan	29	226	1	256
Other	181	4	44	229
**Ethnicity**				
White	896	1	57	954
Hispanic	28	1	373	402
Asian/Pacific Islander	30	229	1	260
Black	5	0	1	6
Multiple/Other	32	2	34	68
**Education (highest qualification)**				
High school	86	24	69	179
Vocational training	86	118	16	220
Bachelor or equivalent	334	96	202	632
Master or equivalent	301	42	142	485
Doctoral	181	52	39	272
**COVID-19**				
Yes	2	1	6	9
Suspected	58	4	16	78
Not sure	143	31	56	230
No	467	87	212	766

In order to evaluate how representative this corpus is of the overall population, it is useful to compare the self-reported demographic data of the corpus with the demographic data provided by the relevant countries. For example, in terms of gender, English-, Japanese-, and Spanish-language respondents self-identified as “female” 74, 54, and 58%, respectively. In comparison, populations in the UK, Japan, and Mexico have female percentages of 51 (19), 53 (20), and 51 (21), respectively. This implies that the English-language part of the corpus has a bias toward female participants, while the Japanese- and Spanish-language parts are more representative. In terms of the age range, the corpus managed to capture the full range of 18–85+ years of all three countries. In addition, the median age of the English-, Japanese-, and Spanish-language respondents was 53, 52, and 40, respectively, which is consistent with median ages in the UK, Japan, and Mexico of 49, 53, and 42 years, respectively (after excluding under-18-year-olds).

Given that participants were self-selected, and also free to answer as much or as little as they preferred, we cannot exclude the possibility that this has introduced other biases into the corpus. Accordingly, caution should be exercised when interpreting the corpus, especially with respect to generalizing findings to the population.

**Figure 1:**
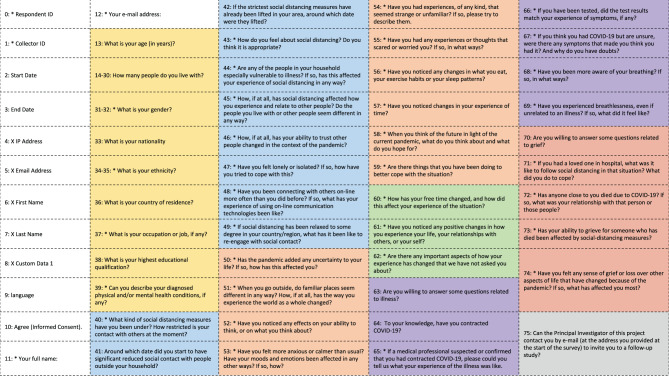
Summary of all the data fields of the “experiences of social distancing during the COVID-19 pandemic” online survey. Fields that are prefixed with a * allowed content that was not restricted to predetermined values.

The survey consisted of six sections with a total of 42 questions (see Figure 1; the English version of the full online survey can be downloaded along with the datasets of the corpus. Participants were free to answer as many questions as they wished. Section 5 and section 6 were optional sections that dealt with more sensitive topics, and these questions were only displayed if participants confirmed their willingness to participate.

- Section 1 (“Personal characteristics”) consisted of nine questions regarding the demographic data and self-described medical history of the participant.
- Section 2 (“Social experience”) consisted of eight open-ended questions about social experience and two questions regarding the date of lockdown measures.
- Section 3 (“General experience”) consisted of 10 open ended questions about general experiences such as the subjective experience of time and space and coping mechanisms.
- Section 4 (“Other experiences”) consisted of three questions about other experiences like the occurrence of any positive changes and hopes for the future.
- Section 5 (“Illness experience”) consisted of six questions related to experiences of illness.
- Section 6 (“Grief experience”) consisted of four questions about the experience of grief.

## 3. Data Description

The corpus is composed of five comma-separated value (CSV) files (EN.csv, JP.csv, JP2EN.csv, ES.csv, ES2EN.csv), where each row contains the answers from a single participant and each column corresponds to a particular question of the survey. Each participant is identified by a unique id which follows the convention *LL*_*CC*_*DDDD* (e.g., *EN*_*UK*_0001), where *LL* stands for language (EN, ES, JP), *CC* country of residence (UK, MX, JP, OO for other, NA not specified), and *DDDD* is an incremental number of the received response based on the starting timestamp.

This is not the place to provide an in-depth analysis of the corpus. We provide a sketch of some of the content that it contains, and which can form the basis for future research. During our initial coding of responses we took a closer look at the UK-resident participants of the corpus (*N* = 760), and we focused on several common themes. In what follows we provide overall percentages of those UK participants that had referred to a particular topic (based on word queries), along with participant IDs of some illustrative examples, including some suitable for future cross-cultural comparisons. Note that the percentages are relatively low when compared to the total number of participants, but they are best considered to represent a minimum number of participants concerned with the topic. This is because participants were not equally descriptive in their responses and they were free to answer only a subset of open questions. In other words, many participants do not get counted in the word searches simply because they did not write many words in response to questions in the first place. Still, a comparison among these percentages reveals some relative tendencies.

### 3.1. Awareness of Breathing

As might be expected, given that COVID-19 is a severe acute respiratory illness, the breath was a topic of concern for this survey. However, perhaps surprisingly, the word “breath” was used relatively rarely; only 9% of UK participants mentioned it (*N* = 70). Among those participants, some reported a higher awareness of their breathing (e.g., EN_UK_0369, EN_MX_0324). Understanding the impact of the virus in the respiratory system was regarded as important for some participants; for example, some even practiced breathing exercises as a helping measure in case of contracting COVID-19 (EN_UK_1044). Others mentioned that their breathing awareness did not change (EN_UK_1375, JP_JP_0106), for instance because it was already heightened due to regular yoga practice (EN_UK_0379) or swimming (EN_UK_0023).

### 3.2. Digital Communication

A notable proportion of participants referred to usage of online and digital communication tools. For instance, “online” was mentioned by 30% of UK participants (*N* = 231), while social media applications (including “WhatsApp,” “Facebook,” “Instagram,” “TikTok,” and “Twitter”) were mentioned by 10% (*N* = 78), and videoconferencing applications (including “Zoom,” “Skype,” and “FaceTime”) were mentioned by 33% (*N* = 252), The use of digital communication mainly involved activities such as attending online religious services (EN_UK_0412); talking to friends and family (EN_UK_0418, EN_MX_0545; EN_JP_0018); online exercise classes, seminars, and conference attendance (EN_OO_0088); remote working (EN_UK_0026); and home school purposes (EN_UK_0165).

### 3.3. Notions of Time

Temporality is a central feature of human experience, and we were interested in how participants experienced time. This topic elicited a lot of responses; “time” was mentioned by 73% of UK participants (*N* = 551). How time was impacted varied between participants. Some participants reported no change of time experience, while others noted changes. In the latter case, there seem to be two distinctive kinds of changes: either people lost their sense of the flow of time, for instance due to boredom (references to “boredom,” “boring,” “bore” were made by 8% of UK participants (*N* = 62), see e.g., EN_UK_0038, EN_MX_0026), or they experienced a more rapid flow of time because they were becoming more productive. For example, one participant mentioned a sense of gaining time because they no longer had to commute to work (EN_OO_0542).

### 3.4. Coping Mechanisms

Participants mentioned coping with crisis and maintaining well-being by engaging in activities such as yoga (UK_EN_0032), meditation (UK_EN_0047), mindfulness (UK_EN_0291), running and cycling (EN_OO_1515), walking the dog (UK_EN_ 0058). Some also report reading books (EN_MX_0619), returning to old hobbies (EN_JP_0309) or starting new ones (EN_JP_2238), talking to friends and family (EN_JP_0623), and spending more time with their families. Finally, many participants reported exercising more, although others had to deal with exercising less.

### 3.5. Interpersonal Relationships

Some participants noted that their sense of trusting others had not changed (EN_UK_0013, EN_MX_ 0573). But other participants reported that trusting neighbors and people in general was now an issue (EN_MX_0663), for instance not trusting others to follow social distancing rules (walking in the street or queuing in the supermarket). There was also notable mistrust in news media and a lack of trust in government decisions (EN_UK_0039). In addition, some people reported a lack of interpersonal contact, which expressed itself as loneliness and sadness (EN_UK_0253; EN_UK_0412).

### 3.6. Experiencing Grief

The section on grief was not concerned specifically with COVID-related bereavements during the pandemic, but with bereavements more generally. The survey was conducted during the early stages of the pandemic, therefore comparatively few people had been experiencing grief due to someone dying of COVID-19 (but see EN_UK_0371). Grief in relation to death was accompanied with a sense of frustration about being unable to travel and attending funerals (UK_EN_0356), and thus there were reports of experiencing grief in isolation instead of as a family process (UK_EN_0027). Other types of grief mentioned were grieving the consequences of the pandemic, especially the inability to meet relatives or friends (UK_EN_0081). Also, there was grief for life before the pandemic, as well as a sense of loss of direction, of meaning, and of opportunities (EN_JP_0298).

### 3.7. Future Hopes

In general terms, participants often thought about future hopes as a return to normality (UK_EN_0050). Many hoped for the arrival of a vaccine (references to “vaccine” or “vaccination” were made by 15% of UK participants, *N* = 116). Others referred to desiring to travel and visit family and friends (UK_EN_0044). Parents mentioned wishing a promising future for their children (EN_JP_0300). Some participants also referred to learning how to be kind to others and exercise positive behavior and human skills (EN_JP_0737). Finally, participants also hoped for political and economic change (UK_EN_0019).

## 4. Future Directions

We are in the process of analysing the corpus using both quantitative and qualitative methods. As a first pass, we are using computational techniques to automatically analyze the written text (also known as “natural language processing” techniques) to provide an overall assessment of language use and valence. On this basis we will select particularly illustrative case studies to guide a deeper phenomenological interpretation of the subjective experience of the pandemic. The novelty of this research approach is that we are thereby integrating methods from the social and computer sciences, allowing us to scale up phenomenological analysis of subjective experience to a large sample. Of particular interest is the cross-cultural aspect of the corpus: this allows investigators to compare whether certain kinds of experiences of the pandemic are shared across cultures, or whether they are context-specific.

The current corpus provides a record of subjective reports for a 2-month period in 2020 when the UK, Japan, and Mexico started to relax the more severe social distancing measures that were in place during the first wave of the pandemic. Now, over a year later, we are undergoing another important transition, as vaccines have started to be administered in these countries. In order to get a sense for the current state of the pandemic experience, we have re-launched the survey for the 1,026 participants who had agreed to be contacted about future studies. We plan to publish the responses we are collecting in future work as another publicly available corpus. This will enable researchers to also perform longitudinal analyses of people's responses, comparing their experience after the initial wave of the pandemic with their experience after the onset of vaccination programs. Given that some systematic reviews of the psychological impact have found minimal effects on symptoms of mental illness (22), and others highlight that most people are psychologically resilient to the effects of lockdowns (6), it will be particularly interesting to take a closer look at the conditions that enabled this resilience. We expect that this will uncover important insights into the nature of human flourishing, even under adverse conditions.

## Data Availability Statement

The datasets presented in this study can be found in a Figshare online repository (10.6084/m9.figshare.15060138) at the following link: https://figshare.com/articles/dataset/_/15060138.

## Ethics Statement

The studies involving human participants were reviewed and approved by Human Subjects Research Review Committee (HSRRC), Okinawa Institute of Science and Technology Graduate University (OIST). The patients/participants provided their written informed consent to participate in this study.

## Author Contributions

TF and MR initiated the project. TF, TM, MR, HC, AM, MB, CH, and FS contributed to the design of this study, including defining the questionnaire, and data collection. FS implemented the questionnaire as an online survey, managed participant recruitment, extracted the corpus, and performed initial natural language processing and statistical analyses. JR performed the initial data coding. TF and JR wrote the first draft of the manuscript. All authors reviewed and finalized the manuscript.

## Conflict of Interest

The authors declare that the research was conducted in the absence of any commercial or financial relationships that could be construed as a potential conflict of interest.

## Publisher's Note

All claims expressed in this article are solely those of the authors and do not necessarily represent those of their affiliated organizations, or those of the publisher, the editors and the reviewers. Any product that may be evaluated in this article, or claim that may be made by its manufacturer, is not guaranteed or endorsed by the publisher.
